# Characterization of microRNAs derived from the HIV-1 TAR RNA hairpin

**DOI:** 10.1186/1742-4690-10-S1-P24

**Published:** 2013-09-19

**Authors:** Alex Harwig, Ben Berkhout, Atze Das

**Affiliations:** 1 Laboratory of Experimental Virology, Department of Medical Microbiology, Center for Infection and Immunity Amsterdam (CINIMA), Academic Medical Center, University of Amsterdam, Amsterdam, The Netherlands

## Background

The transacting responsive (TAR) hairpin is present at the 5’ and 3’ end of the HIV-1 RNA genome. This TAR hairpin is essential for HIV-1 replication because it binds the viral Tat transcriptional activator protein. Several groups have demonstrated the presence of small TAR-derived RNAs in HIV-1 infected cells. Supposedly, these small RNAs are microRNAs (miRNAs) produced by the RNAi pathway. We used the sensitive SOLiD ultra-deep sequencing method for an in-depth characterization of these small TAR miRNAs in HIV-1 expressing cells.

## Materials and methods

293T cells were transfected with plasmids expressing the wild-type HIV-1 genome or doxycycline (dox)-dependent HIV-rtTA variants. In HIV-rtTA, the Tat-TAR transcription mechanism is inactivated through mutations in TAR and functionally replaced by the dox-inducible Tet-On system. We used this special HIV-rtTA construct because it allows partial or complete deletion of TAR and mutational inactivation of Tat. Two days after transfection, small intracellular RNA was purified and analyzed by SOLiD sequencing and northern blotting.

## Results

SOLiD analysis of the RNA from the HIV-1 expressing cells revealed the presence of miRNAs corresponding to both the 5’ and the 3’ side of the TAR stem, with the 3’ fragments being more abundant. The cleavage pattern of the miRNAs differed from the patterns expected for the Drosha/Dicer mediated RNAi pathway. Northern blot analysis of the intracellular RNA confirmed the presence of the TAR miRNAs and their TAR RNA precursor. Analysis of TAR- and Tat-mutated HIV-rtTA variants revealed that these TAR RNAs and miRNAs are produced exclusively from the TAR element present at the 5’ end of the viral transcripts and was not influenced by Tat. This production did not require the activation of transcription by dox, which demonstrates that non-processive transcription at the 5’ LTR is sufficient.

## Conclusions

Non-processive transcription at the 5’ LTR promoter of HIV-1 results in the production of TAR RNAs and TAR-derived miRNAs. The observed cleavage pattern does not correspond with the pattern expected for the Drosha/Dicer mediated RNAi pathway and suggests the involvement of another nuclease.

